# ELDER ABUSE IN OLDER ADULTS WITH DEMENTIA: PROTECTIVE FACTORS AND ADVERSE EFFECTS

**DOI:** 10.1093/geroni/igae098.0062

**Published:** 2024-12-31

**Authors:** Wenxing Wei, sarah Balser, Ann Nguyen, Weidi Qin

**Affiliations:** Case Western Reserve University, Cleveland, Ohio, United States; Case Western Reserve University, Cleveland, Ohio, United States; Case Western Reserve University, Cleveland, Ohio, United States; University of Wisconsin Madison, Madison, Wisconsin, United States

## Abstract

With the rapid increase in the aging population, more attention has been paid to studying older adults with dementia. Despite the fact that older adults with dementia are more likely to be abused compared to their cognitively intact counterparts, little attention has been paid to abuse within this population. It is important to recognize the unique characteristics and vulnerabilities of this specific group. This systematic review, conducted using the PRISMA model, aims to critically examine, evaluate, and synthesize literature on protective factors and adverse effects of elder abuse among individuals with dementia. A search was undertaken using the Ageline, Medline, CINAHL, and PsycINFO databases for peer-reviewed articles published in English up to June 2023. A total of 291 articles were identified by the systematic search, and eight articles were included in the review. The results showed that protective factors related to elder abuse are mainly examined at a perpetrator level, including caregiver-related factors (e.g., caregiver coping strategies), relational factors (e.g., premorbid relationship rewards), and contextual factors (e.g., informal support from family and friends during the COVID-19 pandemic). Adverse effects, specifically an increased risk of various medical conditions and poor medication adherence, were identified but less frequently discussed. This review highlights the need for better support and training for caregivers of older adults with dementia, as well as comprehensive policies to prevent elder abuse. It also calls for future research to find ways to protect these older adults and mitigate the adverse effects of elder abuse.

